# Two Novel Vesicle-Inducing Proteins in Plastids 1 Genes Cloned and Characterized in *Triticum urartu*

**DOI:** 10.1371/journal.pone.0170439

**Published:** 2017-01-19

**Authors:** Fei Gao, Bo Chen, Juan Jiao, Lijia Jia, Cuimin Liu

**Affiliations:** 1 State Key Laboratory of Plant Cell and Chromosome Engineering, Institute of Genetics and Developmental Biology, Chinese Academy of Sciences, Beijing, China; 2 Department of Clinical Laboratory, PLA Army General Hospital, Beijing, PR China; University of California - Davis, UNITED STATES

## Abstract

Vesicle-inducing protein in plastids 1 (Vipp1) is thought to play an important role both in thylakoid biogenesis and chloroplast envelope maintenance during stress. Vipp1 is conserved in photosynthetic organisms and forms a high homo-oligomer complex structure that may help sustain the membrane integrity of chloroplasts. This study cloned two novel *VIPP1* genes from *Triticum urartu* and named them TuVipp1 and TuVipp2. Both proteins shared high identity with the homologous proteins AtVipp1 and CrVipp1. TuVipp1 and TuVipp2 were expressed in various organs of common wheat, and both genes were induced by light and various stress treatments. Purified TuVipp1 and TuVipp2 proteins showed secondary and advanced structures similar to those of the homologous proteins. Similar to AtVipp1, TuVipp1 is a chloroplast target protein. Additionally, TuVipp1 was able to rescue the phenotypes of pale leaves, lethality, and disordered chloroplast structures of *AtVipp1* (-/-) mutant lines. Collectively, our data demonstrate that TuVipp1 and TuVipp2 are functional proteins in chloroplasts in wheat and may be critical for maintaining the chloroplast envelope under stress and membrane biogenesis upon photosynthesis.

## Introduction

Thylakoids are vital inner membrane systems of cyanobacteria and chloroplasts in which the major complexes associated with oxygenic photosynthesis are found. In higher plants, oxygenic photosynthesis complexes are well characterized, but the biogenesis of thylakoids remains obscure. Similar to thylakoid lipids, pigments and proteins are not synthesized in thylakoid membranes; plants develop a series of lipid and protein transport networks in chloroplasts. The biogenesis of thylakoids is a complicated process linked to the synthesis and maintenance of structural substances (such as pigments, proteins, and lipids) and is affected by the environment (including factors such as temperature, light and nutrients) [1–3].

A protein of 30 kDa was first identified in pea chloroplasts located on both the envelope and thylakoid membranes. This protein was suggested as a candidate for transporting substances (such as pigments, proteins, and lipids) to thylakoids [4]. In 2001, Kroll and coworkers cloned the gene encoding this protein in *Arabidopsis* and named it vesicle-inducing protein in plastids 1 (Vipp1) based on the phenotype of the *hcf155* mutant, in which the structures and components of the thylakoid membrane were severely disrupted. The plants lacked vesicle trafficking between the chloroplast envelope and the thylakoids [5–7]. One study recently reported that Vipp1 may participate in the cpTat protein transport system in chloroplasts [8], and another study reported that Vipp1 plays a role in the biogenesis/assembly of the photosynthetic apparatus in the thylakoid membrane by supplying structural lipids [9]. However, the precise roles of Vipp1 in vesicle trafficking and thylakoid formation remain unclear.

Phage shock protein A (PspA) is the closest homolog of Vipp1 in *E*. *coli*. These proteins share significant sequence homology and appear to have similar secondary structures. α-Helices predominate in both Vipp1 and PspA secondary structures, and the difference between PspA and Vipp1 is that Vipp1 contains additional C-terminal acids of variable lengths in different species [10–12]. PspA is greatly induced by different biotic stressors (e.g., filamentous phages and lipid biosynthesis) and abiotic stressors (e.g., heat and hyperosmosis) [13]. PspA can help to sustain the proton motive force of the inner membranes and maintain membrane stability during invasion by filamentous phages and other stresses [14–16]. Under stress conditions, PspA forms large homologous ring-like complexes with molecular masses greater than 1 MDa. PspA homologous complexes subsequently bind to damaged membranes and form lattice-like scaffold structures that can maintain the stability of the damaged membranes [17]. Similar to PspA, Vipp1 appears to form a large homologous ring-like complex that is greater than 1 MDa in both *Arabidopsis* and *Chlamydomonas* [12]. Rod-like structures that include stacks of the ring particles of Vipp1 have also been observed *in vitro* [18,19]. Coincidentally, similar functions have been elucidated. For example, plastid Vipp1 forms large complexes and protects the stability of chloroplast membranes under osmotic stress in *Arabidopsis* [20,21]. In addition, several research groups have reported that Vipp1 may function in response to different stresses, such as heat, cold, salt, and osmotic pressure [20–22], whereas the precise role of Vipp1 in resisting stress remains unknown.

Here, we cloned two novel Vipp1 genes from *Triticum urartu* and named them TuVipp1 and TuVipp2. We found that Vipp1 proteins from different species showed significant homology in primary acid sequences. Approximately 70% of TuVipp1 and TuVipp2 proteins have helical structures. We purified TuVipp1 and TuVipp2 proteins from an *E*. *coli*. expression system *in vitro*. TuVipp1 and TuVipp2 may also form large homologous complexes greater than 1 MD in native gel assays. Both proteins form ring-like or rod-like structures in signal-particle experiments. They also have extra C-terminal acid peptides that are excluded from the large homologous complex. TuVipp1 and TuVipp2 are expressed in various organs of common wheat and are greatly induced by light and various stress treatments. Furthermore, TuVipp1 is mostly located in chloroplasts, which suggests that its functions are similar to those of CrVipp1 and AtVipp1. TuVipp1 can compensate for AtVipp1 to rescue the phenotypes of *vipp1* mutant plants in *Arabidopsis*. According to our results, both TuVipp1 and TuVipp2 are functional genes/proteins and may play important roles in thylakoid formation and stress resistance in common wheat.

## Methods

### Plant material, growth conditions and treatment

*Arabidopsis thaliana* ecotype Columbia-0 and other mutant lines were surface sterilized and plated on Murashige & Skoog (MS) medium in a growth chamber at 23°C under long-day conditions (16-h photoperiod). After 2 weeks, the seedlings were transplanted into soil under controlled greenhouse conditions.

A T-DNA insertion *Arabidopsis* line corresponding to AtVipp1 (name: SAIL_5_F07) was obtained from the European Arabidopsis Stock Center (NASC, Loughborough, UK). The homozygous and heterozygous AtVipp1 mutants (AtVipp1-/- and AtVipp1-/+) were screened by PCR as described by the Salk Institute Genomic Analysis Laboratory (http://signal.salk.edu/T-DNA_Genotyping_Procedure.ppt). PCR was performed using AtVipp1-specific genomic primers and a primer aligning to the T-DNA insert (S1 Table).

### Gene cloning and protein purification

TuVipp1 and TuVipp2 cDNA sequences were obtained based on the sequencing database of *Triticum urartu* [23]. The coding regions of the mature TuVipp1 and TuVipp2 proteins were amplified by PCR from *Triticum urartu* G1812 cDNA (for primer pairs, see S1 Table). The PCR products (TuVipp1, 759 bp; TuVipp2, 777 bp) were digested and ligated into pET-11a (Novagen). After the plasmid was transformed into *E*. *coli*. BL21 (DE3) cells, the proteins were induced using 0.5 mM isopropyl-β-D-galactopyranoside (IPTG) at 16°C overnight. The cells were harvested, suspended in lysis buffer (30 mM Tris-HCl pH 7.5, 30 mM NaCl, 1 mM EDTA) and then lysed by ultrasonication (Sonicator S-4000, Misonix). After centrifuging at 12,000 g at 4°C, the soluble proteins were subjected to a Source 30Q column (55 ml, GE Healthcare). Subsequently, TuVipp1 and TuVipp2 were applied to a Heparin column (5 ml, GE Healthcare) and an S300 column (120 ml, GE Healthcare) in storage buffer (30 mM Tris-HCl pH 7.5, 30 mM NaCl, and 1 mM EDTA). TuVipp1 and TuVipp2 proteins were concentrated and stored at -80°C. Native polyacrylamide gel electrophoresis (PAGE) was used to check the statuses of both proteins according to a published protocol. Next, 10 μg of purified protein was loaded onto a 5–13% gradient colorless native gel. Sodium dodecyl sulfate (SDS)-PAGE was performed as previously described [18]. Protein samples were loaded onto a 15% SDS-PAGE gel. All of the proteins from native-PAGE and SDS-PAGE were stained with coomassie blue.

### Expression assay and western blotting

To analyze the gene expression pattern, various wheat tissues (roots, leaves, flag leaves, and siliques) were collected in the field. For stress treatments, 10-day-old wheat seedlings were treated with 200 mM NaCl for 4 h, 200 mM mannitol for 4 h, or cold temperature (4°C) for 12 h, after which the leaves were collected. For light treatment, following germination, seedlings were grown under dark conditions for 7 days. Then, the seedlings were placed under a light for 5 min, and sample leaves were collected.

Total RNA was extracted from the samples using Trizol reagent (Invitrogen). For real-time PCR, TuVipp1 and TuVipp2 were amplified using specific q-PCR primers (S1 Table), and TaTubulin2 (GenBank, U76745) was used as the control. Real-time PCR was conducted in a total volume of 20 μl (2 μl of the reverse transcription (RT) reactions, 1 μM gene-specific primers, and 10 μl of SYBR Green Master mix (Roche) on a LightCycler 480 II Real-Time PCR machine (Roche) according to the manufacturer’s instructions. RT-PCR was performed using EasyScript One-Step gDNA Removal and cDNA Synthesis SuperMix (Transgene). For semi-quantitative PCR, TuVipp1 and AtVipp1 transcripts were amplified using specific primers (S1 Table) for 30 cycles, and AtActin8 (GenBank, 841347) was partially amplified using specific primers (S1 Table) for 22 cycles. DNA gel electrophoresis was used to visualize the PCR products.

Western blotting procedure was conducted according to our previous work [18]. Serum anti-CrVipp1 was produced in rat at our institute facilities, and serum anti-Actin was purchased from Abmart.

### Plant transformation and identification

To investigate the functions of TuVipp1 *in vivo*, the plasmid pCAMBIA1300-35S::TuVipp1 was constructed and introduced into *AtVipp1*(-/-) mutant lines. Full-length TuVipp1 was amplified using gene-specific primers (S1 Table). Then, the PCR product was digested with NcoI/BamHI and cloned into the binary vector pCAMBIA1300. The plasmid pCAMBIA1300-35S::TuVipp1 was transformed into *Agrobacterium tumefaciens* strain GV3101 and then introduced into the *AtVipp1*(-/-) mutant by the floral-dip method [24]. Transgenic seedlings were screened on MS medium containing 30 μg/ml hygromycin B. To identify the T3 transgene lines in the *AtVipp1*(-/-) mutant background, PCR was performed with both gene-specific primers and a T-DNA insertion primer (S1 Table).

### Circular dichroism

The secondary structures of TuVipp1 and TuVipp2 were analyzed by a Chirascan^™^-Plus CD Spectrometer (Applied Photophysics Ltd.) in 1-mm cuvettes. All proteins were diluted to 0.1 mg/ml with phosphate-buffered saline (PBS) (0.2 g/L KCl, 8 g/L NaCl, 0.2 g/L KH2PO4, and 1.46 g/L Na2HPO4). A UV wavelength scan was performed with a wavelength range from 195 to 260 nm at 20°C. For each protein spectrum, more than three scans were averaged; the row data were deconvoluted using CDNN software (http://bioinformatik.biochemtech.uni-halle.de/cdnn).

### Proteinase digestion assay

The proteinase digestion assay was performed according to our previous publication [18]. First, 2 μg of proteins was added to the reaction buffer (20 mM Tris-HCl pH 8.0 and 50 mM NaCl) with the indicated amount of subtilisin proteinase (0 μg/ml, 0.5 μg/ml, 1 μg/ml, 2 μg/ml, 5 μg/ml, 10 μg/ml and 50 μg/ml) (PS, Sigma). The mixtures reacted for 60 min at 4°C and were stopped with 1 mM phenylmethylsulfonyl fluoride (PMSF). The fractions from the proteinase reactions were resolved by SDS-PAGE with coomassie blue staining.

### Transmission electron microscopy (TEM)

For single-particle work, as previously described [18], 4 μl of purified TuVipp1 or TuVipp2 at a concentration of 0.1 g/L was adsorbed for 1 min onto carbon-coated grids previously hydrophilized by glow discharge. Excess protein sample was then removed from the carbon support by blotting, and the grid was washed rapidly with water prior to being stained with 1% uranyl acetate for approximately 1 min and then air dried. Images were recorded using an OSIS MEGAVIEW G2 (1K x 1K) CCD digital camera system (Olympus, Japan) at an accelerating voltage of 120 keV on an FEI Spirit transmission electron microscope (FEI, Eindhoven, Netherlands).

For ultrathin section samples, 4-week-old leaves of wild-type, mutant and transgenic plants were cut into small pieces (1 mm) and then incubated in 2% glutaraldehyde and 4% paraformaldehyde in 0.1 M sodium phosphate buffer (pH 7.2) at 4°C overnight. The samples were washed five times for 5 min each in 0.1 M sodium phosphate buffer (pH 7.2) at 4°C and then incubated in 2% OsO4 for 2 h at 4°C. After washing with 0.1 M sodium phosphate buffer (pH 7.2) three times for 5 min each, the samples were dehydrated in a graded acetone series of 30%, 50%, 70%, 85%, 95% and then 100% for 10 min each and embedded in Spurr resin according to standard procedures. Ultrathin sections approximately 60 nm thick were made with an ultramicrotome (UC-7; Lecia Inc., Wetzlar, Germany) using a diamond knife. Ultrathin sections were stained with uranyl acetate and then with lead citrate and viewed under TEM (JEOL-1400; JEOL Ltd., Tokyo, Japan).

### Subcellular localization and confocal imaging

For transient expression, the plasmid pCAMBIA2300-35S::TuVipp1-eGFP was constructed. Full-length TuVipp1 was amplified using gene-specific primers (S1 Table). Then, the PCR product was digested with BamHI/SalI and cloned into the vector. The plasmid was transformed into *Agrobacterium tumefaciens* strain GV3101 and then introduced into *Nicotiana benthamiana* leaves with an injection method previously described [25,26]. Protein expression was examined 24–48 h following injection. For confocal imaging, *Nicotiana benthamiana* leaves after injection were cut into pieces of 1 cm × 1 cm and placed under a coverslip. Confocal fluorescent images were collected using a Zeiss LSM 710 confocal microscope. To detect eGFP, an excitation wavelength of 488 nm and 500- to 530-nm emission pass-filters were used. Chlorophyll autofluorescence was detected with 570-nm excitation and 640-nm emission pass-filters [27].

## Results

### Gene cloning and blasting in *T*. *urartu*

**Fig 1:**
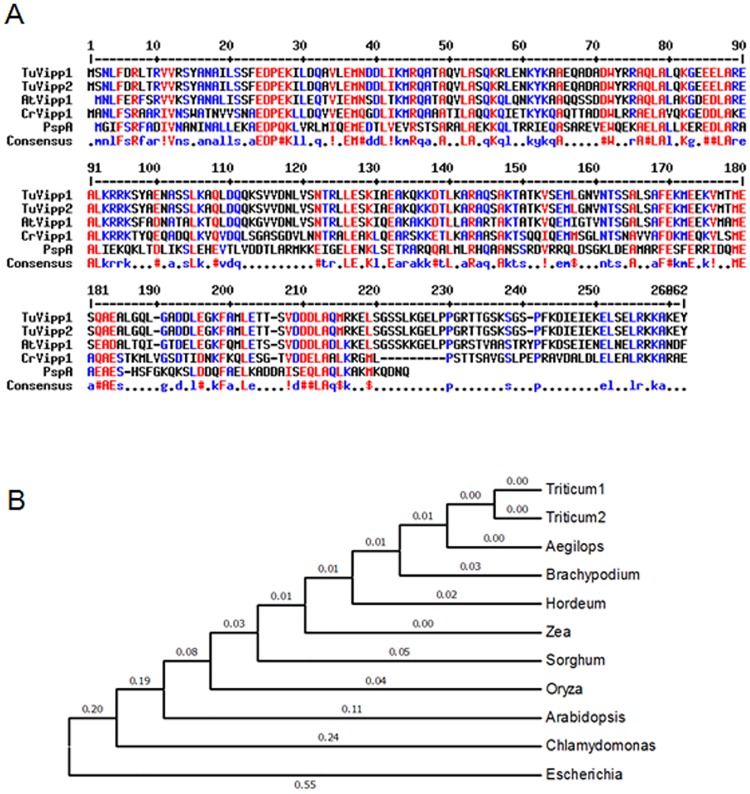
Blasting the sequences of Vipp proteins in different species. (A) Sequence blasting of Vipps from different species. TuVipp1 and TuVipp2 from *Triticum urartu*, AtVipp1 (NP_564846) from *Arabidopsis*, CrVipp1 (XP_001693830) from *Chlamydomonas*, and PspA (WP_000511026) from *Escherichia coli*. (B) Phylogenetic tree of different species. Triticum1, TuVipp1; Triticum2, TuVipp2; Aegilops, Vipp1 (EMT09678) from *Aegilops tauschii*; Brachypodium, Vipp1 (XP_003562483) from *Brachypodium distachyon*; Hordeum, Vipp1 (BAK01930) from *Hordeum vulgare*; Zea, Vipp1 (NP_001130281) from *Zea mays*; Sorghum, Vipp1 (XP_002456776) from *Sorghum bicolor*; Oryza, Vipp1 (EEC71949) from *Oryza sativa*; Arabidopsis, Vipp1 (NP_564846) from *Arabidopsis thaliana*; Chlamydomonas, Vipp1 (XP_001693830) from *Chlamydomonas reinhardtii*; and Escherichia, PspA (WP_000511026) from *Escherichia coli*.

Because Vipp1 functions in thylakoid formation and stress resistance in model plants, we aimed to determine the functions of Vipp1 in crops such as wheat. No novel Vipp1 genes were found in a GenBank BLAST search. We conducted another search using genomic sequences of *Triticum urartu* [23], and two genes were identified. We named these two new genes *TuVIPP1* and *TuVIPP2*. According to sequence information, PCR primers were designed (as shown in S1 Table), and the full-length genes encoding the mature TuVipp1 and TuVipp2 proteins (without signal peptides, shown in S1 Fig) were cloned from *Triticum urartu*. According to sequence blasting, TuVipp1 and TuVipp2 had an approximately 90% sequence identity and had high sequence similarity to the homologous proteins from *E*. *coli*. to higher plant which have been widely reported (Fig 1A). The primary amino acid sequence of the N terminus of Vipp1 is conserved from *E*. *coli*. to higher plants, while that of the C terminus varies. This result suggests that the C terminus is highly differentiated and is linked to specific functions in different species. In a phylogenetic assay, all Vipp1s from monocots were clustered together within a genetic distance of 0.04. TuVipp1 and TuVipp2 clustered in one branch, and the other Vipp1 proteins were related to various species ranging from *E*. *coli*. to higher plants (Fig 1B). This result indicates that TuVipp1 and TuVipp2 co-evolved in *Triticum urartu* and that both proteins may have similar biological functions.

### Expression patterns of TuVipp1 and TuVipp2 in *T*. *urartu*

**Fig 2:**
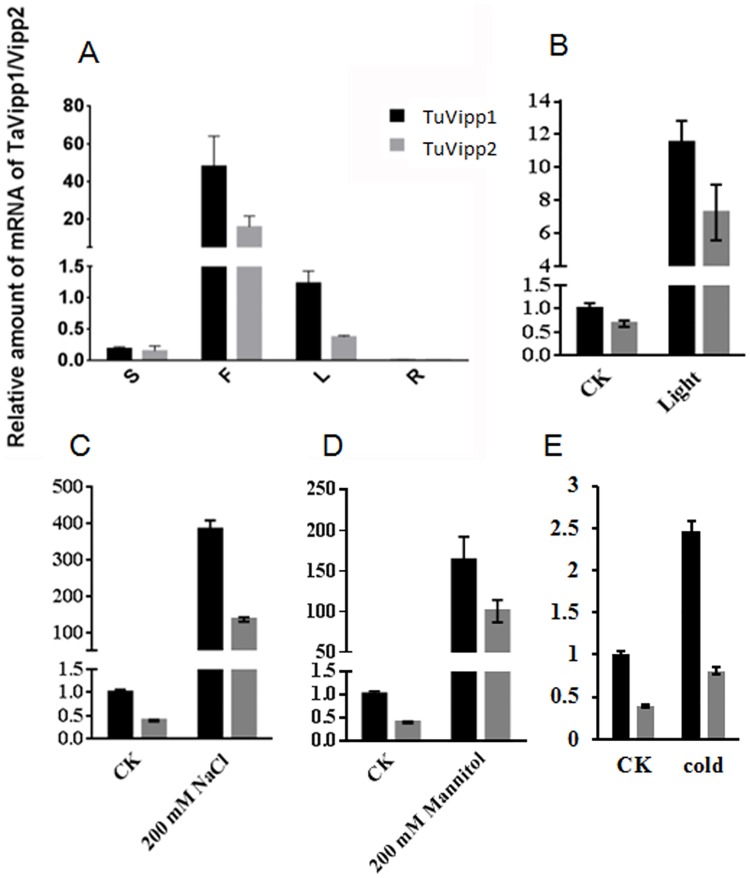
Expression patterns of TuVipp1 and TuVipp2 in wheat. (A) Expression patterns of TuVipp1 and TuVipp2 in different organs. S, siliques; F, flag leaves; L, leaves; R, roots. (B) mRNA levels of TuVipp1 and TuVipp2 after placing the seedlings under light for 5 min. (C-E) mRNA levels of TuVipp1 and TuVipp2 after treatment with 200 mM NaCl_2_, 200 mM Mannitol, or cold temperature (4°C), respectively.

To characterize the TuVipp1/2 expression patterns in wheat, q-PCR was used to check the mRNA levels of TuVipp1 and TuVipp2 in different organs. No TuVipp1 or TuVipp2 mRNA was detected in roots; however, the expression level of TuVipp1 and TuVipp2 mRNA in flag leaves was approximately 40-fold higher than that in shoots and leaves (Fig 2A). This result suggests that TuVipp1 and TuVipp2 are closely linked to photosynthesis. In addition, the mRNA levels of TuVipp1 and TuVipp2 in response to different conditions were also analyzed in wheat. TuVipp1 and TuVipp2 were largely induced from dark to light, and their expression increased more than 10-fold within 5 min of light illumination (Fig 2B). After treating with salt and mannitol for 4 h, TuVipp1 and TuVipp2 were induced at a rate more than 100 times greater than that found under normal conditions (Fig 2C and 2D). Under cold conditions, as shown in Fig 2E, TuVipp1 and TuVipp2 mRNA levels increased 2.5-fold after 12 h of treatment. We also examined the protein levels of TuVipps (TuVipp1 and TuVipp2 together) using a polyclonal antibody and found that TuVipps proteins were accumulated under different stress conditions (data shown in S2 Fig). TuVipp1 and TuVipp2 had similar expression patterns in wheat and may together be involved in resistance to stress conditions. This result agrees with previous reports [20–22].

### Subcellular localization of TuVipp1 and TuVipp2

**Fig 3:**
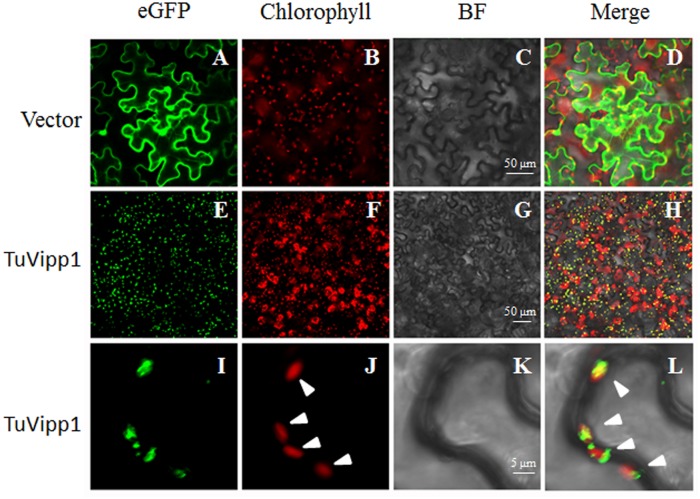
Subcellular localization of TuVipp1. Confocal micrographs showing the chloroplast targeting of TuVipp1. Tobacco leaves transformed with 35S::GFP (A-D) and 35S::TuVipp1-GFP (E-L) plasmids are shown. Chlorophyll autofluorescence (Chlorophyll), eGFP fluorescence (eGFP), bright field (BF), and merged images are shown. The triangles in J and L indicate the chloroplast.

Next, the subcellular localization of TuVipp1 was detected by confocal microscopy. Because the genomic sequence database of *Triticum urartu* [23] has many gaps, we could not obtain information on TuVipp2 signal peptides. Therefore, only full-length TuVipp1 containing signal peptide was constructed in a pCAMBIA vector linked to eGFP and transformed into *Agrobacterium*. Forty-eight hours after being introduced into tobacco leaves, fluorescent signals were observed under a microscope, as shown in Fig 3. Fig 3A, 3B, 3C and 3D show the empty vector; green fluorescence represents eGFP, while red fluorescence represents chlorophyll, which indicates the sites of chloroplasts. This result showed that GFP protein was located throughout all tobacco epidermal cells containing control plasmids. TuVipp1, indicated by green fluorescence, presented a continuous stellate distribution (Fig 3E), and the merge view showed that TuVipp1-GFP and chlorophyll co-localized and exhibited yellow fluorescence (Fig 3H). To further support this result, enlarged pictures are shown in Fig 3I, 3J, 3K and 3L, which shows four chloroplasts (Fig 3J) and the TuVipp1 protein located in or around the chloroplasts (Fig 3L). Collectively, our data show that TuVipp1 is a functional protein in chloroplasts with functional signal peptides. This result agrees with Aseeva’s results in *Arabidopsis* [12].

### Purification of TuVipp1 and TuVipp2 *in vitro*

**Fig 4:**
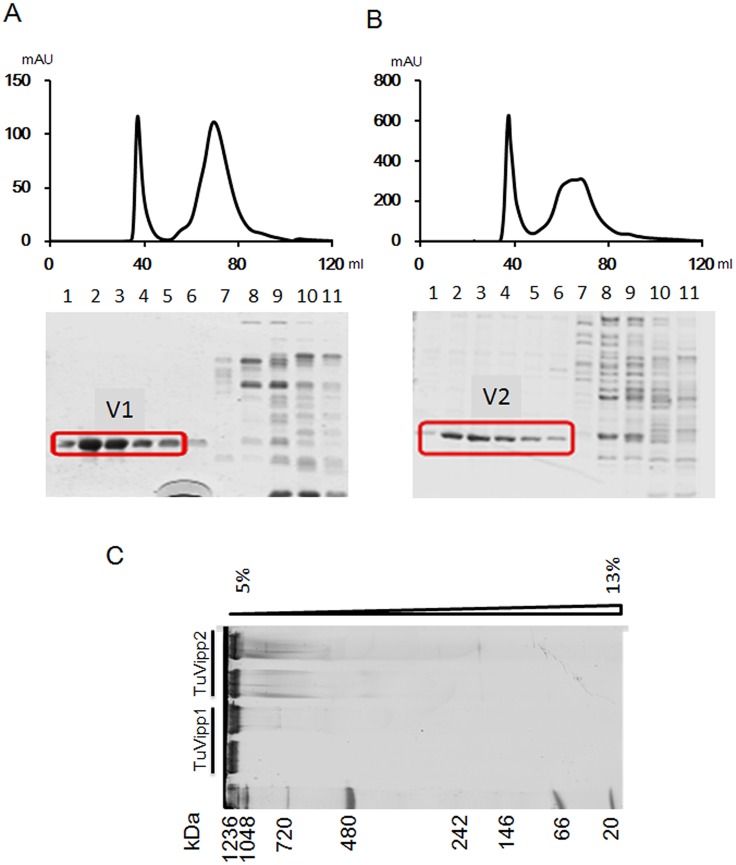
Purification of TuVipp1 and TuVipp2 *in vitro*. (A) The TuVipp1 protein pool was separated on a gel filtration column (S-300, GE), and the eluted fractions of 36–48 ml (band 1 to 6) and 50–90 ml (band 7 to 11) were separated by 12% SDS PAGE. X axis, volume from gel filtration column; Y axis, absorbance intensity of A280. (B) The TuVipp2 protein pool was separated on a gel filtration column (S-300, GE), and the eluted fractions of 36–48 ml (band 1 to 6) and 50–90 ml (band 7 to 11) were separated by 12% SDS PAGE. X axis, volume from gel filtration column; Y axis, absorbance intensity of A280. (C) Analysis of TuVipp1 and TuVipp2 in a colorless gradient native gel (5%-13%). The marker is a commercial marker (Cat LC0725, Thermo Fisher).

To study the properties of TuVipp1 and TuVipp2, both proteins (without signal peptides) were constructed in a pET11a vector and expressed in an *E*. *coli*. system. Both proteins were induced by IPTG and pre-purified with a Source 30Q column without any detergent and then subjected to a S300 column. TuVipp1 and TuVipp2 were eluted at approximately the void volume (Fig 4A and 4B, 36–48 ml) from the S300 column. This result suggests that TuVipp1 and TuVipp2 have a molecular weight of approximately 1 MDa. Another peak at A280 occurred due to other unknown proteins (Fig 4A and 4B, 50–90 ml). In addition, a native gel was used to check the statuses of TuVipp1 and TuVipp2, as shown in Fig 4C. Both proteins exhibited one major band that barely entered the gel, with a molecular weight of more than 1.2 MDa. TuVipp1 and TuVipp2 may form a high-oligomeric complex larger than 1 MDa *in vitro*, which agrees with the sizes of Vipp1 previously reported in *Arabidopsis* and *Chlamydomonas* [12,18,19,28].

### Chemical and structural properties of the TuVipp1 and TuVipp2 proteins

**Fig 5:**
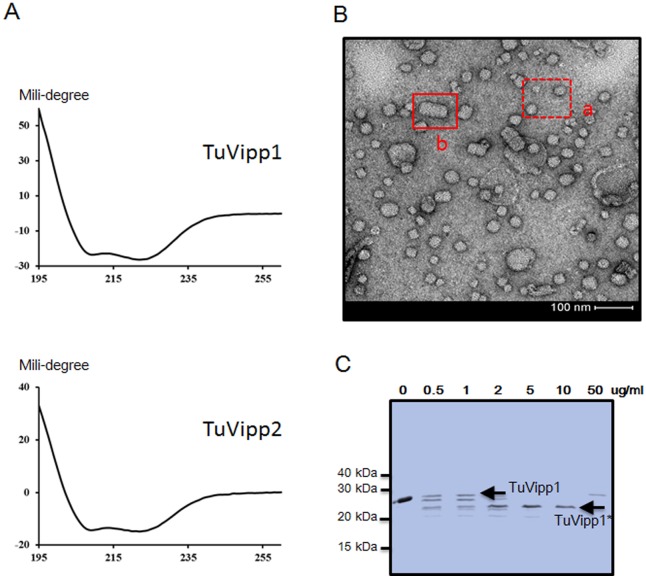
Structural analyses of TuVipp1 and TuVipp2 *in vitro*. (A) Circular dichroism spectroscopy curve of purified TuVipp1 and TuVipp2 proteins. Y-axis values represent the raw data from a Chirascan^™^-plus CD Spectrometer. (B) Transmission electron micrograph images of full-length TuVipp1. a shows the “ring-like” structures of TuVipp1, and b shows the “rod-like” structures of TuVipp1. The white bar represents a scale of 100 nm. (C) Proteinase protection experiment. TuVipp1 treated with subtilisin proteinase at different concentrations (0, 0.5, 1, 2, 5, 10 and 50 μg/ml). 'TuVipp1' indicates full-length TuVipp1 (approximately 29 kDa), and 'TuVipp1*' (approximately 25 kDa) denotes the major product from the digestion.

To study the secondary structures of TuVipp1 and TuVipp2, these two proteins were subjected to circular dichroism spectroscopy. After being deconvoluted by CDNN software, 63% of CrVipp1 and 68% of CrVipp2 were found to have helical structures; random coil or other secondary structures occurred in less than 40% of the proteins (Fig 5A, Table 1). In addition, the ratios of molar ellipticity at 208 to 222 nm were 0.85 (TuVipp1) and 0.94 (TuVipp2), indicating that both proteins may form coiled-coil structures (Table 1). The secondary structures of TuVipp1 and TuVipp2 were also predicted by the program JPred 3. As shown in S3 Fig, both proteins had 8 α-helical structures interrupted by random coils, and coiled-coil structures were found in the third α-helical domain. These features were similar to those of the homologous proteins in *Arabidopsis* and *Chlamydomonas* [18,28].

**Table 1:** Content of the helical structure in both TuVipp1 and TuVipp2 proteins.

	TuVipp1	TuVipp2
Helix	63.90%	68.80%
Antiparallel	1.60%	1.30%
Parallel	3.90%	3.30%
Beta-Turn	12.00%	11.50%
Rndm. Coil	18.60%	15.20%
208 nm/222 nm	0.86	0.94

In a higher structure assay, single-particle characteristics were observed. As with AtVipp1 and CrVipp1, both TuVipp1 and TuVipp2 were able to form “ring” structures with different diameters or packed rod-like structures (Fig 5B). This result confirmed our previous results [18,28]. As previously reported, compared with PspA in *E*. *coli*., Vipp1 proteins from *Arabidopsis* and *Chlamydomonas* have a C-terminal extension that extrudes from a highly ordered polymer structure [11,12,18,28]. To assess the flexibility of the C-terminus of TuVipp1, a protease protection experiment was conducted. As shown in Fig 5C, after cleavage by the non-specific proteinase subtilisin at different concentrations, one stable fragment of approximately 25 kDa was produced. Q-TOF mass spectrometry was used to identify this stable fragment of TuVipp1 and showed that a small fragment of peptide (approximately 38 aa) at the C terminus was digested by the proteinase (S4 Fig). This result indicates that TuVipp1 also has a flexible C terminus that extrudes from its high polymeric complex.

### The functions of TuVipp1 in vivo

**Fig 6:**
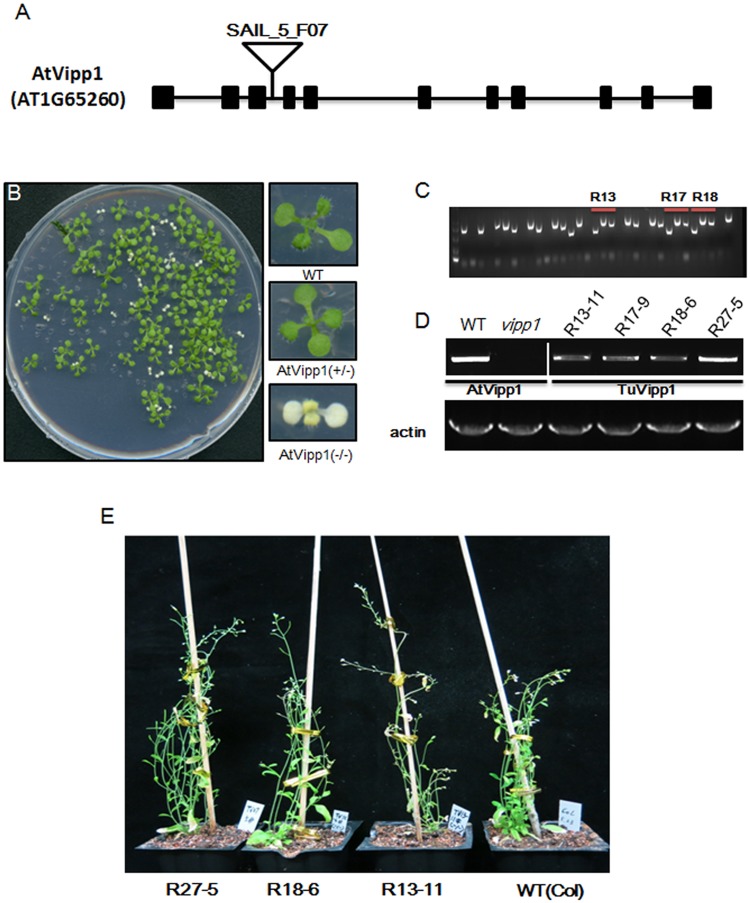
Functional analysis of TuVipp1. (A) The intron/exon organization of the AtVipp1 gene (AT1G65260) and the location of the T-DNA inserted into the third intron of the AtVipp1 gene in the SAIL_5_F07 mutant line. Black boxes, exons; black lines, introns; black triangle, T-DNA insertion site. (B) The phenotype of the mutant (SAIL_5_F07) of the AtVipp1 gene (AT1G65260). Left large picture showed a seedling of the heterozygous mutant of SAIL_5_F07. The seedling showed two phenotypes (right small picture): heterozygous mutant seedlings (AtVipp1 +/-) showed a WT phenotype, while homozygous mutant seedlings (AtVipp1 -/-) showed A pale and dwarf phenotype. (C) Screening the transgene lines of TuVipp1 in the homozygous mutant (SAIL_5_F07) background of AtVipp1 by PCR. Each line contains three PCR reactions with different primer pairs (TuVipp1-specific primers, T-DNA left border primer and AtVipp1-specific primers). TuVipp1-specific primers amplify the TuVipp1 gene in transgene lines; T-DNA left border primer and AtVipp1-specific primers amplify the AtVipp1 gene in the homozygous mutant background (described in method 2.1). R13, R17 and R18 are the T2 transgene lines of TuVipp1 with the heterozygous mutant of AtVipp1. (D) The expression of TuVipp1 in T3 transgene lines of TuVipp1 in the homozygous mutant (SAIL_5_F07) background of AtVipp1 by semi-quantitative PCR. WT, amplifying AtVipp1 mRNA as a positive control; *vipp1*, amplifying AtVipp1 mRNA as a negative control; R13-11, R17-9, R18-6, and R27-5, amplifying TuVipp1 mRNA. The AtActin8 gene was used as a control gene. (E) The phenotypes of the T3 transgene lines of TuVipp1 in the homozygous mutant (SAIL_5_F07) background of AtVipp1. R13-11, R18-6, and R27-5 are T3 transgene lines of TuVipp1 in the homozygous mutant (SAIL_5_F07) background of AtVipp1 and showed a similar phenotype as that of wild type plants.

One *Arabidopsis* T-DNA insertion mutant line (Sail_5_F07) was used to verify the biological functions of TuVipp1. In these mutants, the T-DNA is inserted in the third intron of the AtVipp1 gene (AT1G65260), as shown in Fig 6A. As shown in Fig 6B, the homozygous T-DNA-insertion AtVipp1 mutants had a serious abnormal phenotype; the plants were pale and dwarf. However, the phenotype of the heterozygous mutants was similar to that of the wild type. These phenotypes were similar to those of other AtVipp1 mutants, including *hcf155*, for example [7]. We therefore transformed the full-length TuVipp1 with signal peptides into the heterozygous AtVipp1 mutants. PCR was used to screen the plants containing the homozygous trans-TuVipp1 gene and T-DNA insertion lines. For example, R13, R17 and R18 were heterozygous T-DNAs inserted at the AtVipp1 locus, and TuVipp1 was successfully transformed into the heterozygous mutants (Fig 6C). Then, semi-quantitative PCR showed that the mRNA levels of TuVipp1 in the T3 transgene lines of R13-11, R17-9, R18-6 and R27-5 (Fig 6D) were rescued to the levels of wild-type plants. Finally, we examined the phenotype of rescue lines, as shown in Fig 6E. TuVipp1 was able to fully rescue the pale and dwarf phenotypes caused by the deletion of AtVipp1 in transgene lines R27-5, R18-6 and R13-11.

**Fig 7:**
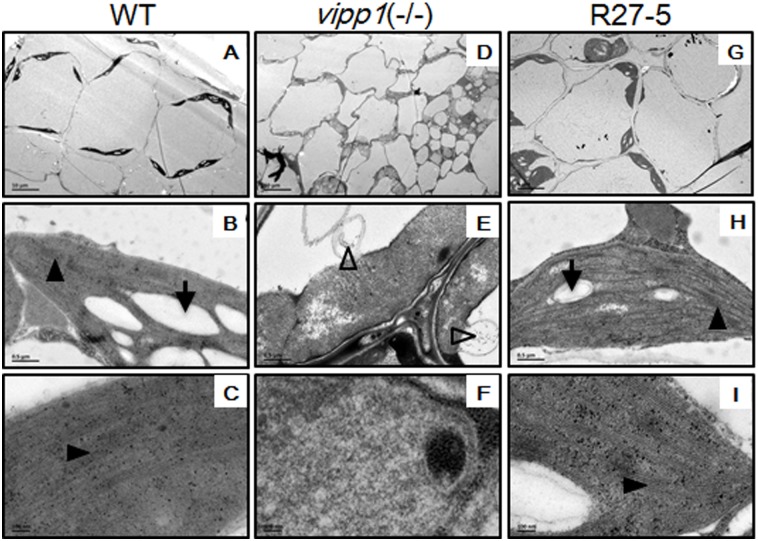
Electron microscope images of chloroplast structure in different plants. (A-C) The wild-type chloroplast structures under different magnifications. (D-F) The chloroplast structures of the homozygous mutant of AtVipp1 (AtVipp1-/-) under different magnifications. (G-I) The chloroplast structures of the rescue plant (R27-5) in a homozygous mutant background (AtVipp1-/-) under different magnifications. The arrow shows the starch granule, the black triangle shows the thylakoid membrane, and the hollow triangle shows the balloon structure. The black bar represents the magnification scale.

Previous research reported that the structure of the thylakoid membrane and vesicular transport were abolished in AtVipp1 mutants [7]. These phenotypes were also observed in homozygous AtVipp1 Sail_5_F07 mutants (Fig 7F) in electron microscopy experiments. In contrast, the Sail_5_F07 mutant plants had abnormal chloroplasts (Fig 7D), starch granules (Fig 7E), and thylakoid structures (Fig 7F). In the rescue transgenic line R27-5, the structures of the chloroplasts, starch granules and thylakoids were similar to those of wild-type plants (Fig 7A, 7B 7G, 7H and 7I). In addition, the homozygous AtVipp1 mutant exhibited special balloon structures at the edges of the chloroplasts that protected the chloroplasts from osmotic stress, as previously reported [20,21]. This phenotype may be related to the function of Vipp1 in stress resistance.

## Discussion

Vipp1 (M30) was first identified on both the envelope and thylakoid membranes of pea chloroplasts, which suggested that it was a transport protein for carrying substances from the envelope to thylakoids [4]. In 2001, Kroll and coworkers first cloned Vipp1 in *Arabidopsis*. The structures and components of the thylakoid membrane were severely disrupted in AtVipp1 mutants (*hcf155*). The vesicle trafficking between the chloroplast envelopes and the thylakoids also diminished [7]. Subsequently, Vipp1 was found to be closely linked to thylakoid formation and vesicle trafficking, but the precise roles of Vipp1 in this process remained unclear. During the following decade, many reports suggested that Vipp1 was able to form a ring-like, large homo-oligomer complex with a molecular mass of more than 1 MDa. Vipp1 was also shown to have an extra C terminus excluded from the homo-oligomer complex. In addition, Vipp1 was shown to take part in different stress responses, such as responses to osmotic pressure, heat, cold, and salt [20–22]. Most recently, Wataru Sakamoto and coworkers illuminated the functions of the C-terminal tail in protecting photosynthetic membranes against stress [21], but the mechanism by which Vipp1 regulates plant stress resistance (such as resistance to osmotic pressure, cold, and salt) remains unknown.

Fortunately, Vipp1 has a homologous gene in *E*. *coli*: phage shock protein A (PspA). Vipp1 and PspA share significant sequence homology and appear to have similar secondary and advanced structures. Therefore, the study of PspA may help uncover new clues regarding the precise roles of Vipp1 in stress resistance. In our study, we were interested in the roles of Vipp1 in stress resistance and thylakoid formation in crops such as wheat. Because the genome of common wheat is complex, two Vipp1 homolog genes were cloned according to the sequencing database of *Triticum urartu* [23] and named TuVipp1 and TuVipp2. The TuVipp1 and TuVipp2 proteins have many similar properties: 1) They have high primary protein sequence identification (Fig 1A). 2) They co-evolved during wheat evolution (Fig 1B). 3) The expression patterns of TuVipp1 and TuVipp2 are very similar in various tissues under normal conditions and under different stress treatments (Fig 2). 4) The properties of the two proteins are also very similar; for example, these proteins form a high homo-oligomer complex, and the composition of their secondary structures and their ring-like structure formation are similar (Fig 5, some data not shown). It has been suggested that a gene rearrangement and doubling event may have occurred during the evolution of TuVipp1 in wheat. Therefore, we used TuVipp1 to analyze gene function in wheat. First, we investigated the subcellular localization of TuVipp1 *in vivo*. Fluorescent images showed that TuVipp1 is located in chloroplasts (Fig 3), confirming that TuVipp1 is a chloroplast-targeted protein. This result agrees with previous reports on *Arabidopsis* [12]. We also assessed the biological functions of TuVipp1 in *Arabidopsis*. Plants that were generated by transforming the TuVipp1 gene into AtVipp1 (-/-) mutants showed a rescue of the pale and lethality phenotypes of the AtVipp1 (-/-) lines (Fig 6B and 6E). This result was further supported by analyzing the chloroplast structures in ultrathin sections of leaves from rescued plants and AtVipp1 (-/-) mutants. In rescued plants, the chloroplast structures were normal, as in wild-type plants (Fig 7). Notably, we observed a balloon-like structure at the edges of the chloroplasts in AtVipp1 (-/-) mutant lines under normal conditions. This balloon-like structure was also observed under osmotic stress in *Arabidopsis* [20,21]. Vipp1 likely has a universal function in chloroplast envelope maintenance under both normal and stress conditions. In our work, TuVipp1 and TuVipp2 were first cloned and studied in monocot plants. Our research will open the door for studying the regulation of photosynthesis, membrane biosynthesis and stress resistance in monocot plants, especially in crops.

## Supporting Information

S1 Fig Protein sequences of TuVipp1 and TuVipp2. The primary acid sequences of TuVipp1 and TuVipp2, which were cloned in *Triticum urartu*.
(TIFF)

S2 Fig Protein levels of TuVipps (TuVipp1 and TuVipp2 together) in wheat. Protein levels of TuVipps detected by western blot. (A) Accumulation of TuVipps proteins in different organs. S, siliques; F, flag leaves; L, leaves; R, roots. (B) Accumulation of TuVipps proteins treating with 200 mM NaCl_2_ (4h), 200 mM Mannitol (4h), or cold temperature (4°C, 12h), respectively. (C) Accumulation of TuVipps proteins after placing the seedlings under light for 4h and 24h. Actin and Rubisco were used as internal control.
(TIFF)

S3 Fig Predictions of helical structures of TuVipp1 and TuVipp2 by JPred 3. Both TuVipp1 and TuVipp2 have seven a-helices, and the third a-helix may form coiled-coil structures. H indicated a-helix.
(TIFF)

S4 Fig Protease treated TuVipp1 detected by LC-MS/MS. Red letters indicated the detected fragments. The C terminal 223–259 aa is not detected in the protease treated TuVipp1.
(TIFF)

S1 Table Primer List. Primer list for gene cloning, gene expression and screening transgene plants.
(TIFF)
