# Supplementary figures and images for: Two Novel Vesicle-Inducing Proteins in Plastids 1 Genes Cloned and Characterized in Triticum urartu
Source: PLoS One. 2017 Jan 19;12(1):e0170439. doi: 10.1371/journal.pone.0170439 (PMC5245824; doi:10.1371/journal.pone.0170439)

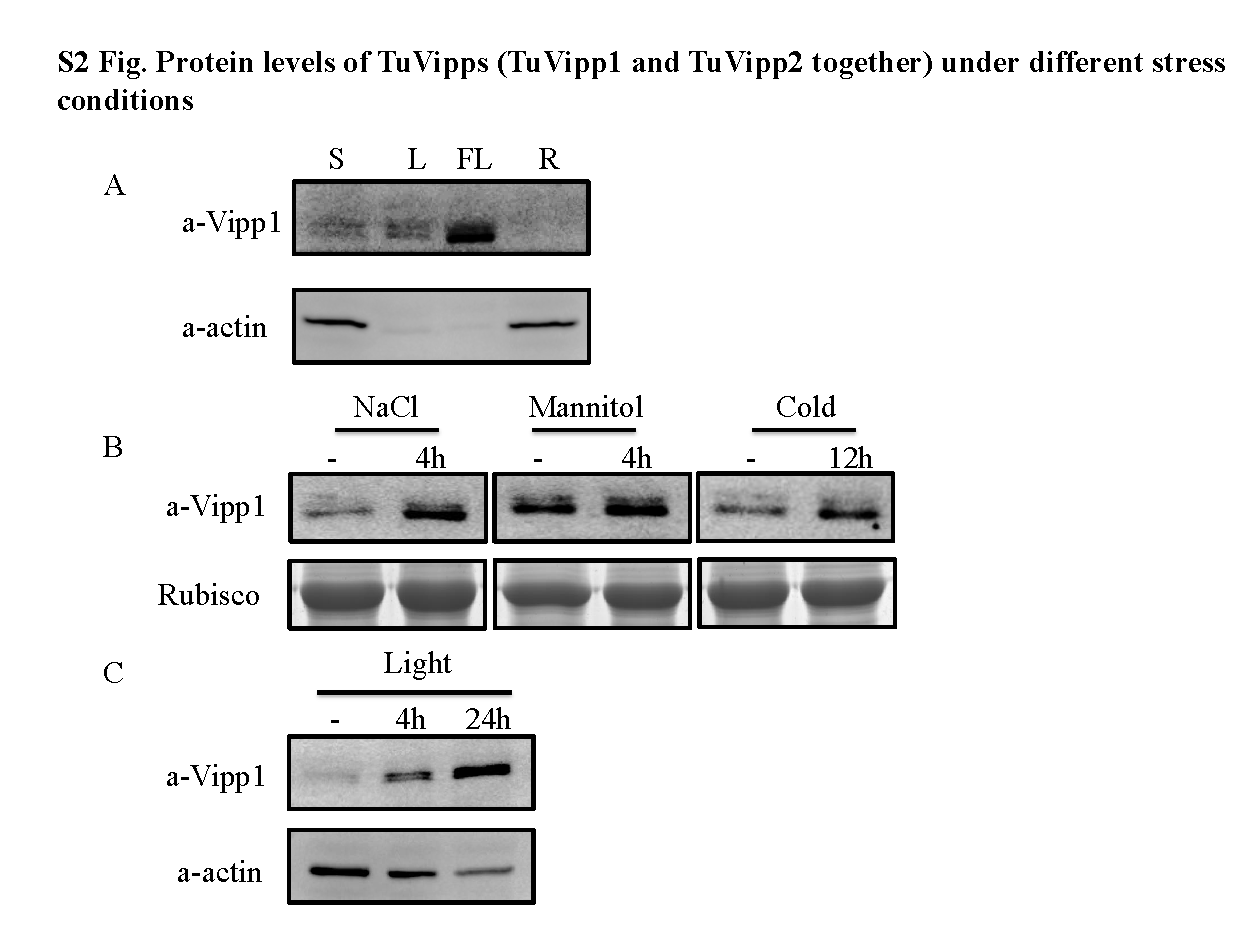

Supplement: S2 Fig — Protein levels of TuVipps detected by western blot. (A) Accumulation of TuVipps proteins in different organs. S, siliques; F, flag leaves; L, leaves; R, roots. (B) Accumulation of TuVipps proteins treating with 200 mM NaCl2 (4h), 200 mM Mannitol (4h), or cold temperature (4°C, 12h), respectively. (C) Accumulation of TuVipps proteins after placing the seedlings under light for 4h and 24h. Actin and Rubisco were used as internal control. (TIFF) [file pone.0170439.s002.tiff]

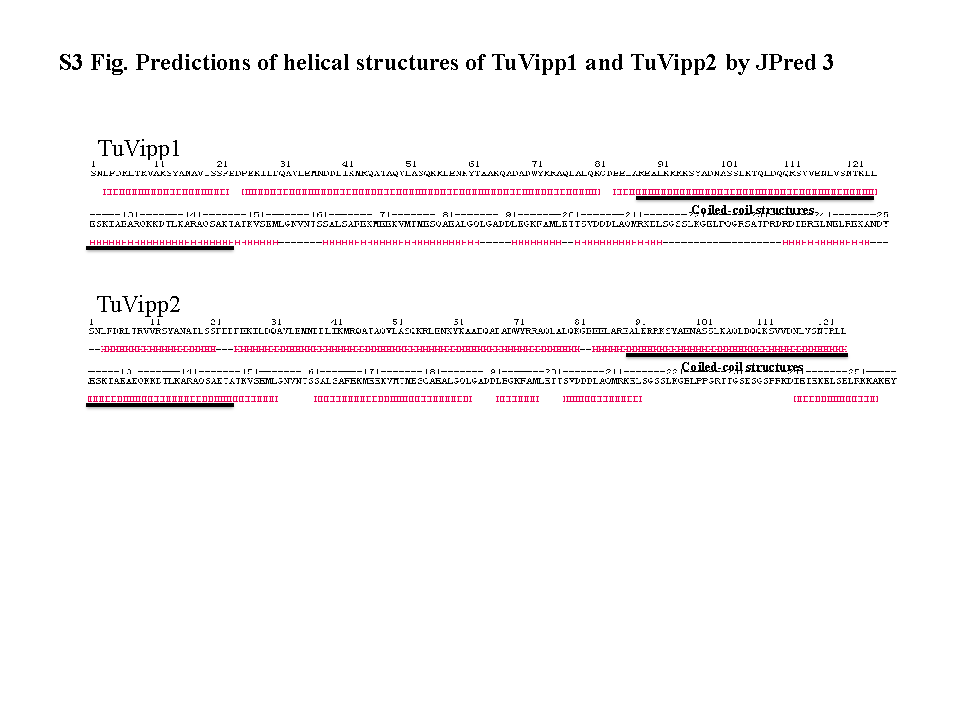

Supplement: S3 Fig — Both TuVipp1 and TuVipp2 have seven a-helices, and the third a-helix may form coiled-coil structures. H indicated a-helix. (TIFF) [file pone.0170439.s003.tiff]

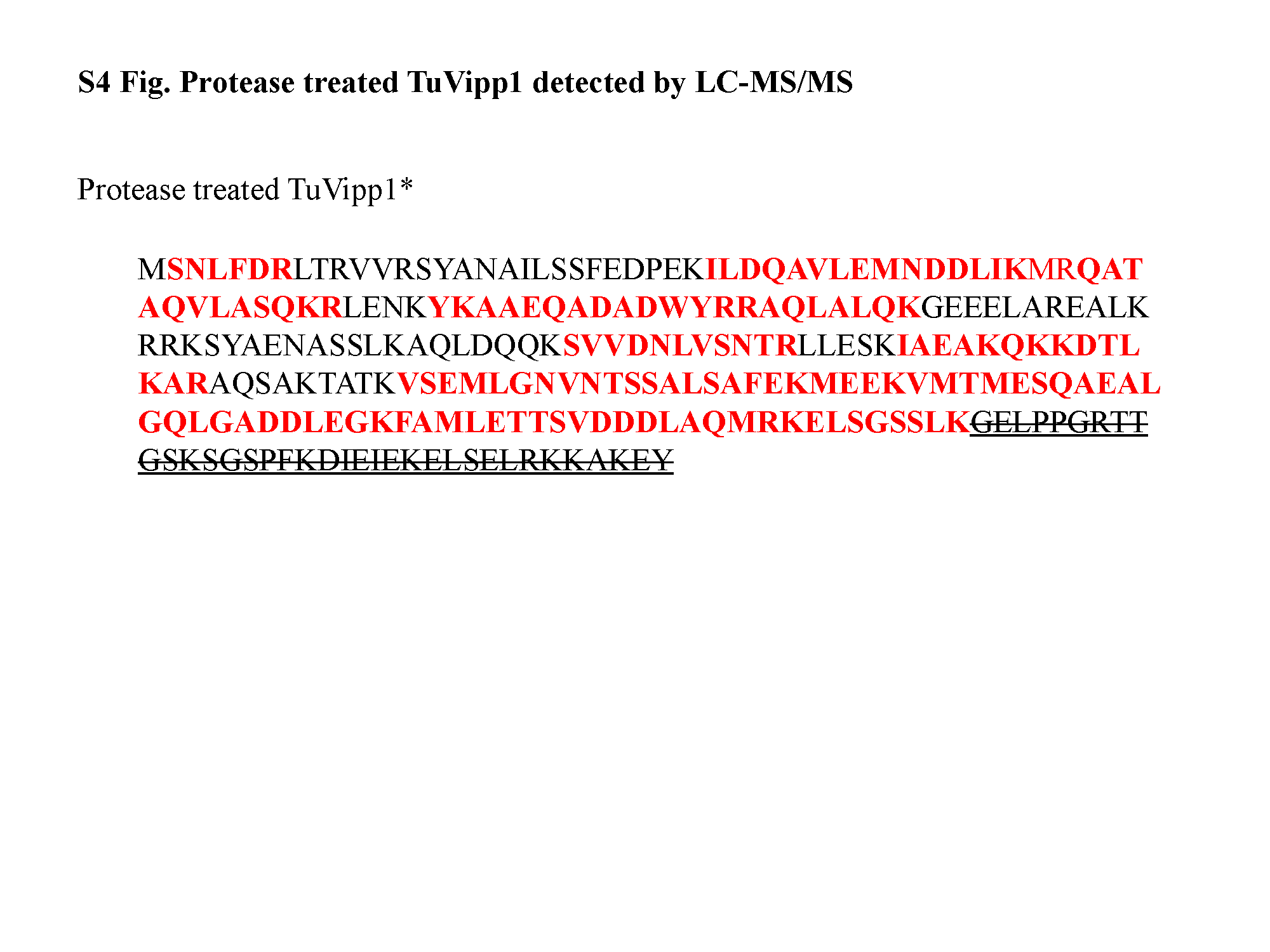

Supplement: S4 Fig — Red letters indicated the detected fragments. The C terminal 223–259 aa is not detected in the protease treated TuVipp1. (TIFF) [file pone.0170439.s004.tiff]
